# Orbital emphysema as a rare complication of asthma exacerbation in a pediatric patient; A case report

**DOI:** 10.1016/j.ijscr.2025.111441

**Published:** 2025-05-14

**Authors:** Akihiro Ichiki, Keisuke Takata, Ichiro Hamasaki, Tadashi Moriwake

**Affiliations:** a Department of Pediatrics, Iwakuni Clinical Center, Yamaguchi, Japan; b Lino Eye Clinic, Japan; c Department of Ophthalmology, Iwakuni Clinical Center, Yamaguchi, Japan

**Keywords:** Orbital emphysema, Spontaneous pneumomediastinum, Subcutaneous emphysema, Asthma, Case report

## Abstract

**Introduction:**

Spontaneous pneumomediastinum (SPM) and subcutaneous emphysema (SCE) are well-known complications of asthma. Orbital emphysema (OE) is a rare complication, with little known of its pathogenesis and clinical significance. This report presents a case of OE associated with asthma exacerbation in a pediatric patient.

**Presentation of case:**

An 8-year-old girl with a history of asthma treatment developed wheezing and periorbital swelling around her right eye following influenza A infection. She had no history of trauma, nose blowing, or recent surgery. Head and chest computed tomography revealed SPM, massive SCE, and right OE. Intraocular pressure and visual acuity were normal. OE, SCE, and SPM were completely resolved through conservative management, without any sequelae.

**Discussion:**

OE may be associated with asthma exacerbation in pediatric patients. We hypothesized that the massive SCE and SPM, which developed as a result of asthma exacerbation, spread through the fascial planes, leading to the development of OE.

**Conclusion:**

OE is typically a benign self-limiting condition; however, it may lead to serious complications such as orbital compartment syndrome. Clinicians should consider OE in patients with asthma who present with sudden orbital symptoms, even in the absence of a history of trauma.

## Highlights

• Orbital emphysema was identified as a rare complication of asthma exacerbation.
• This is the first case report of orbital emphysema following influenza infection.
• This case provides valuable insights into the pathogenesis of orbital emphysema.

## 1 Introduction

Spontaneous pneumomediastinum (SPM) and subcutaneous emphysema (SCE) are well-known complications of asthma [1]. In contrast, orbital emphysema (OE) most commonly results from fractures of the orbital walls and lacerations of the adjacent sinus mucosa and is rarely recognized as a complication of asthma [2], though its pathogenesis and clinical significance remain largely unclear. Herein, we report a case of OE, SPM, and SCE following acute exacerbation of asthma and influenza A infection in the absence of trauma.

This work has been reported in accordance with the SCARE guidelines [3].

## 2 Presentation of case

**Fig. 1:**
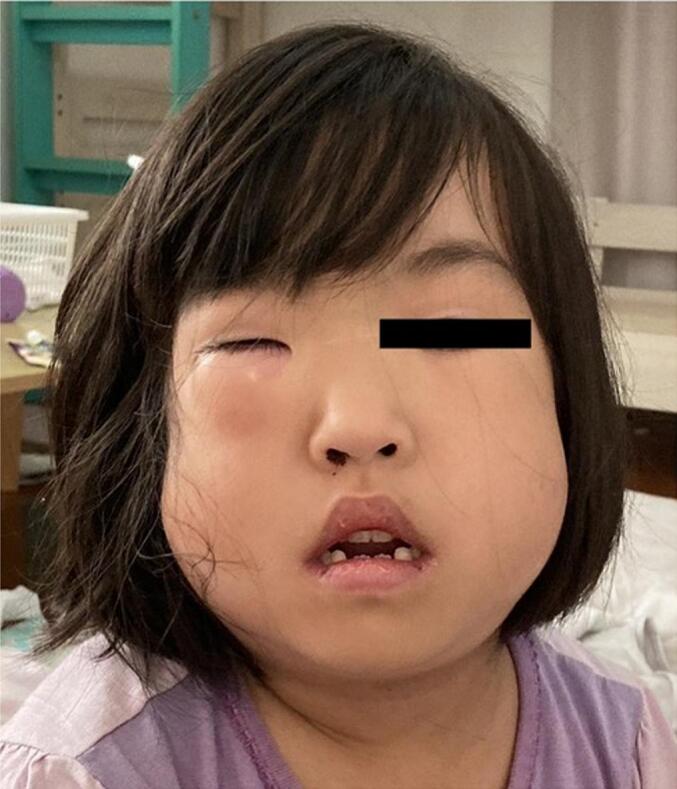
Periorbital swelling of the right eye in a female patient.

**Fig. 2:**
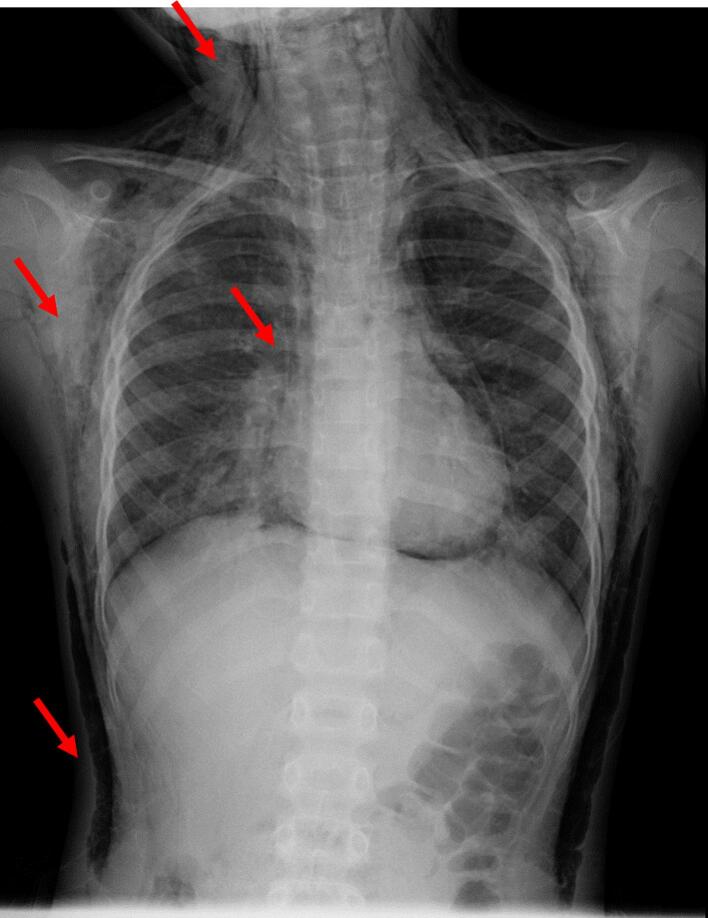
A frontal chest radiograph showing pneumomediastinum (red arrows in the middle) and subcutaneous emphysemas (red arrows in the upper and lower chest).

**Fig. 3:**
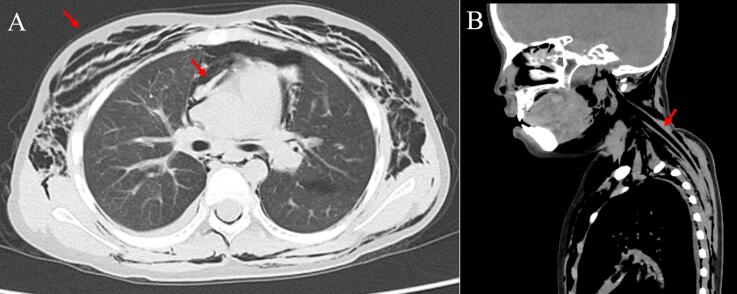
A. Axial chest computed tomography showing subcutaneous emphysema (red arrow) and pneumomediastinum (black arrow). B. Computed tomography (CT) showing a continuous distribution of air from the mediastinum to the cervical soft tissue.

**Fig. 4:**
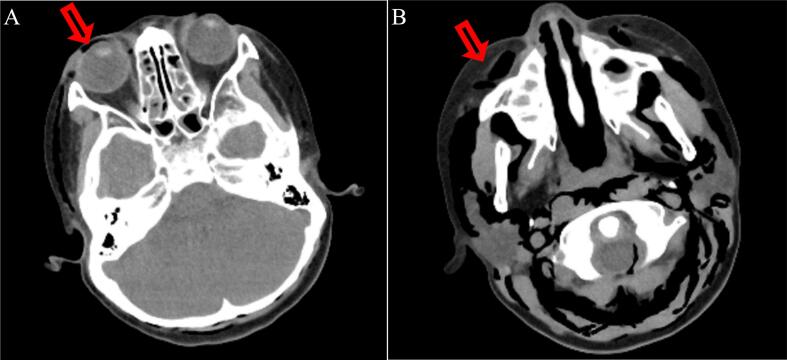
A. Axial computed tomography of facial bones showing orbital emphysema (red arrow). B. Axial computed tomography of facial bones showing subcutaneous periorbital emphysema (red arrow).

An 8-year-old female was referred to our hospital by her primary care physician with a 2-days history of fever, cough, and shortness of breath. On the day of admission, the patient developed swelling around the right eye and cheek (Fig. 1). She had a history of asthma and sinusitis treatment but no history of trauma, nose blowing, or recent surgery. She had normal growth and development and received all routine childhood vaccinations. No known drug or food allergies were detected. On initial physical examination, she had swelling around the right orbit and cheek, diffuse wheezing with a markedly prolonged expiratory phase in all lung fields, orthopnea, and difficulty speaking. The patient had no rashes on her face or trunk. Her body mass index (BMI) was 15.3 (height: 117 cm, weight: 21 kg), classifying her as underweight. She had a respiratory rate of 30 breaths/min, heart rate of 140 beats/min, blood pressure of 110/60 mmHg, temperature of 38.0 °C, and oxygen saturation of 90 % on ambient air. We initially considered anaphylaxis to have caused the wheezing and facial swelling, though intramuscular epinephrine injection did not improve her respiratory condition. Blood tests revealed elevated white blood cell counts (14.7 × 10 [3]/μL) with neutrophilia (83.3 % of total cell count) and elevated C-reactive protein levels (7.3 mg/dL). Chest radiography revealed SPM with massive SCE in the neck and trunk without a pneumothorax (Fig. 2). Computed tomography (CT) revealed SPM and SCE extending from the neck to the trunk and enhanced interstitial marking in the right lung field (Fig. 3). CT of the head revealed SCE of the face, right OE, and sinusitis, with no evidence of blowout fracture (Fig. 4). Nucleic acid testing of the nasopharyngeal specimen for SARS-COV-2 was negative, and rapid antigen testing for influenza type A was positive. During the second physical examination, she complained of chest pain and snowball crepitations around her face, neck, and trunk. On initial examination, the ophthalmologist noted swelling, erythema and tenderness in both the upper and lower eyelids of the right eye. The patient did not report any visual disturbances, and there was no noticeable difference in visual perception between the right and left eyes. Intraocular pressure was within the normal range, and no substantial signs of severe orbital compression were noted. To differentiate orbital emphysema from other etiologies, a head magnetic resonance imaging (MRI) was performed. The head MRI showed no increase in the fat tissue density around the orbit. We diagnosed OE, SPM, and SCE associated with asthma. She underwent conservative treatment, receiving an inhaled β2-agonist and intravenous glucocorticoids for asthma, intravenous peramivir for influenza A, and prophylactic intravenous sulbactam–ampicillin. At the ophthalmological follow-up one week after the initial visit, the patient showed marked clinical improvement. Given the absence of abnormal findings at that time, repeated orbital CT imaging was considered unnecessary and therefore not performed. After 10 days, the OE, SCE, and SPM were completely resolved without sequelae, and the patient was discharged.

## 3 Discussion

Around 99.6 % of OE cases are associated with orbital fractures [4]. Its occurrence following an asthma exacerbation is extremely rare. To the best of our knowledge, only one case of OE secondary to acute asthma exacerbation has been reported [5], and this is the first case to provide a detailed anatomical mechanism.

In acute severe asthma, increased airway resistance, high ventilatory demand, and shortened expiratory time prevent full exhalation, leading to lung hyperinflation. This creates an intrinsic positive end-expiratory pressure (PEEP). The elevated intra-alveolar pressure caused by intrinsic PEEP can lead to alveolar rupture. Air leakage from alveolar ruptures into the surrounding broncho-vascular sheath resulting in SPM and SCE, known as the Macklin effect [6].

Maunder et al. described to a continuity of fascial planes extending from the mediastinum to cervical soft tissues [7]. Although their report did not specifically refer to the orbit or periorbital region, similar air tracking pattern has been described in case reports of OE following laparoscopic surgery [8], Nissen fundoplication [9], and thoracotomy [10]. These cases suggested that air can spread through contiguous fascial planes toward the orbit. Although the underlying mechanisms differ from asthma, these cases share the anatomical pathway that supports the plausibility of orbital air dissection through fascial planes. In our case, CT demonstrated a continuous distribution of air extending from the mediastinum to the cervical region and into the right orbit. Furthermore, there was a clear temporal relationship between the onset of asthma symptoms and the development of orbital emphysema. These finding support the anatomical and clinical possibility of asthma-induced OE. Further investigation is required to confirm these findings and identify the risks associated specifically with asthma.

OE has been reported in association with Valsalva maneuvers, which can be triggered by activities such as nose blowing [11], weightlifting [12], or sneezing [13], as well as surgical procedures, including retinal [14] and endoscopic sinus surgery [15]. In our case, based on parental report, the patient had not engaged in any Valsalva-inducing activities, including forceful coughing or nose blowing, prior to symptom onset. There was no history of facial trauma or relevant surgical procedures. Therefore, as the patient had neither a history of Valsalva-inducing activities nor relevant surgical procedures, both causes were considered unlikely. This supports the interpretation that asthma-related barotrauma was the most likely etiology in this case.

The differential diagnosis of OE can further be explained by infection with gas-producing organisms and foreign bodies. In cases of trauma, it is essential to consider concurrent injuries. OE has been associated with intracranial emphysema [16], and CT scans, including intracranial scans, are useful for detecting the presence, position, and volume of air.

OE is a benign self-limiting condition that usually resolves without intervention. However, serious complications such as orbital compartment syndrome, central retinal artery occlusion, and compressive optic neuropathy have been reported [16,17]. Typical signs of OE include periorbital subcutaneous crepitus, proptosis, decreased vision, relative afferent pupillary defects, and subconjunctival emphysema [2]. Management strategies for OE depend on its severity; mild cases are typically only observed, moderate cases may require lateral canthotomy and needle decompression, and severe cases often require urgent surgical intervention [2]. Therefore, prompt evaluation by an ophthalmologist is crucial to prevent irreversible vision loss in suspected cases.

## 4 Conclusion

OE may be a rare complication of asthma exacerbation. Although OE is typically self-limiting, complications such as orbital compartment syndrome may require invasive treatment by an ophthalmologist and are associated with a poorer prognosis. Clinicians should consider OE in patients with asthma who present with sudden orbital symptoms, even in the absence of historical trauma.

## Abbreviations

SPM: Spontaneous pneumomediastinum
SCE: Subcutaneous emphysema
OE: Orbital emphysema
BMI: Body Mass index
CT: Computed tomography
MRI: Magnetic Resonance Imaging
PEEP: positive end-expiratory pressure

## Consent

Written informed consent was obtained from the patient and parents for the publication of this case report and accompanying images. In addition, age-appropriate explanations were provided to the 8-year-old patient, who assented to the use of her clinical information. A copy of the form is available for review upon request.

## Ethical approval

Not applicable.

## Guarantor

Akihiro Ichiki.

## Funding

This research did not receive any specific grant from funding agencies in the public, commercial, or not-for-profit sectors.

## Author contribution

AI wrote the manuscript.

IH and KT were involved in managing the patient.

TM supervised and approved the manuscript.

## Declaration of competing interest

The authors declare that there are no conflicts of interest regarding the publication of this case report.
